# Factors associated with an increased risk of developing pneumonia during acute ischemic stroke hospitalization

**DOI:** 10.1371/journal.pone.0296938

**Published:** 2024-01-10

**Authors:** Pornpong Jitpratoom, Adhiratha Boonyasiri

**Affiliations:** 1 Division of Medicine, Chumphon Khet Udomsakdi Hospital, Chumphon, Thailand; 2 Faculty of Medicine Siriraj Hospital, Department of Research and Development, Mahidol University, Bangkok, Thailand; CHU Nantes, FRANCE

## Abstract

Stroke-associated pneumonia (SAP) is a common complication of acute ischemic stroke (AIS). This single-center retrospective observational study aimed to identify factors associated with SAP and predictors of poor outcomes in hospitalized patients with AIS. The study included patients admitted to Chumphon Khet Udomsakdi Hospital in Thailand within 7 days of the onset of AIS between July 2019 and July 2020. The patients were divided according to whether they were diagnosed with SAP during hospitalization into a pneumonia group and a non-pneumonia (control) group. Factors associated with SAP were identified. After 3 months, the patients with AIS were divided into those with a poor outcome (modified Rankin scale [mRS] score ≥4) and those with a non-poor outcome (mRS score <4). Factors associated with a poor outcome were sought. During the study period, 342 patients (mean age 65 years, 61% men) were admitted with AIS, of whom 54 (15.8%) developed SAP. Multivariate analysis identified a failed water-swallowing test (WST; adjusted odds ratio [aOR] 87.48, 95% confidence interval [CI] 21.00–364.51, p<0.001), endotracheal intubation with invasive mechanical ventilation (aOR 12.38, 95% CI 2.44–101.35, p = 0.001), and a retained Foley catheter (aOR 5.67, 95% CI 2.03–15.83, p = 0.001) to be associated with SAP. Of the 342 patients, 112 (32.7%) had a poor outcome at 3 months, predictors of which included having hypertension as a comorbidity (aOR 2.87, 95% CI 1.18–6.98, p = 0.020), a pre-stroke mRS score ≥2 (aOR 4.53, 95% CI 1.50–12.72, p = 0.007), an initial Barthel Index score <40 (aOR 3.35, 95% CI 1.57–7.16, p = 0.002), a failed WST (aOR 5.04, 95% CI 2.00–12.74, p = 0.001), and brain edema (aOR 20.67, 95% CI 2.10–203.26, p = 0.009). This study emphasized the association of SAP with a failed WST, endotracheal intubation with invasive mechanical ventilation, and a retained Foley catheter but also identified hypertension, a pre-stroke mRS score ≥2, an initial BI score <40, a failed WST, and brain edema as predictors of a poor outcome for patients 3 months after AIS.

## Introduction

Stroke-associated pneumonia (SAP) is a common infectious complication of stroke and has variously been reported to occur in 1.2%–22% of patients admitted to hospital with acute stroke [1]. SAP typically has a poor clinical outcome, including an increased risk of mortality and long-term disability [2,3]. Several factors increase the risk of respiratory tract infection in patients with acute ischemic stroke (AIS). These may be a direct consequence of the brain injury caused by the stroke; for example, dysphagia, an impaired cough reflex, and immobility all increase the risk of aspiration [1]. Accumulating evidence also suggests that brain-induced immunosuppression increases the risk of systemic infections in stroke patients via the central nervous system-mediated impairment of immune competence [4–6]. However, these infections could also be an indirect consequence of factors associated with the stroke but not caused by it, such as advanced age and comorbidity [1]. Although data on SAP are limited [7] and not entirely consistent, predictors appear to include severe hypertension, older age (>65 years), pre-stroke disability, speech impairment, dysphagia, tube feeding, tracheal intubation, and comorbidity (in particular, chronic obstructive pulmonary disease, coronary artery disease, and diabetes) [1,4–6,8–11]. This study aimed to identify associated factors for SAP in patients with AIS during hospitalization and predictors of poor outcomes in patients with AIS 3 months after diagnosis.

## Materials and methods

This retrospective observational study was performed at Chumphon Khet Udomsakdi Hospital, a 509-bed regional teaching hospital that provides clinical services in Chumphon Province, Thailand. The data were accessed for research purposes on 1^st^ October 2020. Patients aged 18 years or older who were admitted with a diagnosis of AIS within 7 days of symptom onset between July 2019 and July 2020 were screened for eligibility for inclusion in the study. However, due to the coronavirus disease 2019 outbreak in Thailand that began in March 2020, every patient prior to admission and inpatients with fever or respiratory symptoms had been screened. If their test results were positive, they were not included. Patients for whom clinical and laboratory data, chest radiographs, brain images, and 3-month outcome data were incomplete were also excluded. Pneumonia was diagnosed based on the presence of at least three of the five following acute lower respiratory tract symptoms and signs: fever (temperature ≥37.8°C), cough, dyspnea (respiratory rate >25 breaths/minute), breathing-related (pleuritic) chest pain, and signs of consolidation or crackles combined with a chest radiograph showing evidence of new infiltration [12–17]. Eligible patients were then divided according to whether or not they developed pneumonia within 7 days of AIS onset during hospitalization and classified based on their meeting the SAP criteria of the Pneumonia in Stroke Consensus Group [18] into a pneumonia group and a non-pneumonia (control) group. Pneumonia that developed 48 hours or more after admission to the hospital was classified as hospital-acquired pneumonia (HAP) [19,20], and pneumonia that was present before 48 hours was classified as community-acquired pneumonia (CAP). Patients who developed pneumonia after endotracheal intubation for more than 48 hours were defined as having ventilator-associated pneumonia (VAP) [21–23]. Pneumonia that developed sequentially after macroaspiration within 3 days was diagnosed as aspiration pneumonia [24].

Baseline information was collected on demographics and clinical characteristics, including age, sex, and comorbidities. The following AIS data on admission were also recorded: presenting symptoms; results of initial laboratory investigations; Glasgow Coma Scale score (used for the objective determination of the extent of impaired consciousness, ranging from 3 [coma] to 15 [normal]) [25,26]; National Institutes of Health Stroke Scale (NIHSS) score (used for the objective quantification of stroke severity, ranging from 0 [normal] to 42 [coma with quadriplegia]) [27,28]; Trial of Org 10172 in Acute Stroke Treatment (TOAST) classification [29]; modified Rankin Scale (mRS) score (representing degree of disability because of stroke and ranging from 0 [no symptoms] to 6 [death]) [30]; and the Barthel Index (BI) score (used to measure performance in activities of daily living, ranging from 0 [totally dependent] to 100 [normal]) [31]. The 3-month outcome was assessed using the mRS and BI scores, whose use for this purpose is supported by substantial evidence [30,31].

Information on pneumonia was collected, including signs and symptoms, chest radiographs, pathogens, antibiotic sensitivity, and antibacterial treatment. The CURB-65 score and Systemic Inflammatory Response Syndrome (SIRS) criteria for identifying sepsis were also explored. The CURB-65 score is a pneumonia severity score that comprises five variables, with one point assigned for each of the following: new-onset confusion; urea >19 mg/dL. respiratory rate ≥30/min, systolic blood pressure <90 mmHg and/or diastolic blood pressure ≤60 mmHg, and age ≥65 years [32]. This score has been extensively validated as a predictor of 30-day mortality in patients with pneumonia [33]. The SIRS criteria require at least two of the following: tachycardia (heart rate >90 beats/min); tachypnea (respiratory rate >20 breaths/min); fever or hypothermia (temperature >38°C or <36°C); and leukocytosis, leukopenia, or bandemia (white blood cells >12,000/mm^3^ or <4,000/mm^3^, or band count >10%) [34]. Invasive and non-invasive mechanical ventilation, endotracheal intubation, nasogastric tube placement, Foley catheter status, and water-swallowing test (WST) results were also reviewed.

The data were compared between the pneumonia group and the control group to identify factors associated with pneumonia. The impact of pneumonia on the results of treatment, including post-stroke complications, length of stay, status at discharge, and 3-month mRS and BI scores, was also investigated. For stroke complications, progressive stroke was defined as the gradual worsening of neurological function (NIHSS score increase ≥4) during the 72 hours after stroke onset from an ongoing ischemic process [35–40]; brain edema was diagnosed when the patient had a new neurological deficit from brain swelling that was seen in a brain image [41]; and symptomatic intracranial hemorrhage was defined as any intracranial hemorrhage with neurologic deterioration, as indicated by an NIHSS score of ≥4 points higher than the baseline value [42]. The characteristics of all patients who developed SAP were examined in detail. Finally, determinants of a poor outcome (mRS score ≥4) after 3 months in patients with AIS were sought.

### Statistical analysis

All statistical analyses were performed using the PASW Statistics 18.0 package (Predictive Analytics Software, SPSS Inc., Chicago, IL, USA). Descriptive statistics were used to summarize demographic variables, including patient age and sex. Quantitative data were presented as the mean ± standard deviation or median (interquartile range) and qualitative data as the frequency (percentage). Differences in categorical variables (e.g., patient sex) were compared between the pneumonia and control groups using the chi-squared test or Fisher’s exact test. Differences in quantitative variables (e.g., patient age) were compared between the two groups using the independent *t*-test or Mann–Whitney *U* test. Variables with a p-value <0.05 in univariate analysis were considered for entry in multivariate analysis. Multiple logistic regression was used to estimate adjusted odds ratios (aORs) and 95% confidence intervals (CIs) in the pneumonia and control groups using a backward method with a probability of removal of 0.17. Categorical variables were compared between the patients with AIS according to whether the 3-month outcome was poor (mRS score ≥4) or non-poor (mRS score <4) using the chi-squared test or Fisher’s exact test. All factors with a p-value of <0.05 in univariable analysis were considered for inclusion in multivariable analysis using multiple logistic regression (with the backward step method), and their aORs and 95% CIs were calculated to compare the data according to whether or not the outcome was poor.

The study was approved by the human research ethics committee of the Faculty of Medicine, Thammasat University (Ref: MTU-EC-OO-0-180/63). The requirement for informed consent was waived by the ethics committee owing to the retrospective observational nature of the research. All methods were performed in accordance with the relevant guidelines and regulations, including the Declaration of Helsinki, the Belmont Report, the Council for International Organizations of Medical Sciences guidelines, and ICH-Good Clinical Practice guidelines.

## Results

### Demographic data

A total of 342 patients hospitalized for AIS during the study period were enrolled. Their mean age was 65±15 years, and more than half (61%) of the patients were male. According to the TOAST classification, AIS was caused by small-vessel occlusion in 158 patients (46%), followed by large-artery atherosclerosis in 104 (30%), cardioembolism in 66 (19%), other determined etiology in 11 (3%), and undetermined etiology in three (1.0%). The five most common comorbidities were hypertension (72%), dyslipidemia (54%), diabetes mellitus (30%), chronic kidney disease (23%), and atrial fibrillation (18%). Furthermore, 41% of the patients smoked, 36% were obese (body mass index ≥25, calculated as kg/m^2^), 25% consumed alcohol, and 2% had chronic obstructive pulmonary disease.

### Factors associated with an increased risk of developing SAP

Pneumonia was diagnosed in 54 patients (15.8%), who were assigned to the pneumonia group. The remaining 288 patients without pneumonia were assigned to the control group. The results of the univariate analysis of factors potentially associated with pneumonia are shown in **Table 1** and those of the multivariate analysis in **Table 2.** Multivariate analysis identified SAP to be associated with a failed WST (aOR 87.48, 95% CI 21.00–364.51, p<0.001), endotracheal intubation with invasive mechanical ventilation (aOR 12.38, 95% CI 2.44–101.35, p = 0.01), and a retained Foley catheter (aOR 5.67, 95% CI 2.03–15.83, p = 0.001).

**Table 1:** Factors identified to be potentially associated with stroke-associated pneumonia via univariate analysis.

Variable	Total (n = 342)	Control group (n = 288)	Pneumonia group (n = 54)	p-value*
Sex				0.92
Male, n (%)	207 (60.5)	174 (60.4)	33 (61.1)	
Female, n (%)	135 (39.5)	114 (39.6)	21 (38.9)	
Age, mean ± SD (years)	65±15	64±15	73±14	<0.001
Age ≥70 years, n (%)	141 (41.2)	105 (36.5)	36 (66.7)	<0.001
BMI, mean ± SD	24±5	24±5	24±4	0.71
Obesity (BMI ≥25), n (%)	122 (35.7)	102 (35.4)	20 (37.0)	0.82
**Lifestyle habits**				
Alcohol consumption, n (%)	87 (25.4)	80 (27.8)	7 (13.0)	0.022
Smoking, n (%)	140 (40.9)	125 (43.4)	15 (27.8)	0.032
**Comorbidities**				
Hypertension, n (%)	246 (71.9)	207 (71.9)	39 (72.2)	0.96
Diabetes mellitus, n (%)	101 (29.5)	86 (29.9)	15 (27.8)	0.76
Chronic kidney disease, n (%)	79 (23.1)	59 (20.5)	20 (37.0)	0.008
Dyslipidemia, n (%)	185 (54.1)	156 (54.2)	29 (53.7)	0.95
Coronary artery disease, n (%)	42 (12.3)	28 (9.7)	14 (25.9)	<0.001
Atrial fibrillation, n (%)	62 (18.1)	42 (14.6)	20 (37.0)	<0.001
Previous stroke, n (%)	52 (15.2)	43 (14.9)	9 (16.7)	0.74
BPH (men only), N (%)	9 (2.6)	5 (1.7)	4 (7.4)	0.038
COPD, n (%)	7 (2.0)	6 (2.1)	1 (1.9)	1.00
HIV infection, n (%)	2 (0.6)	2 (0.7)	0 (0.0)	1.00
**Presenting symptoms**				
Alteration of consciousness, n (%)	59 (17.3)	32 (11.1)	27 (50.0)	<0.001
Headache, n (%)	25 (7.3)	19 (6.6)	6 (11.1)	0.25
Weakness, n (%)	303 (88.6)	250 (86.8)	53 (98.1)	0.016
Facial palsy, n (%)	91 (26.6)	75 (26.0)	16 (29.6)	0.58
Visual disturbance, n (%)	35 (10.2)	34 (11.8)	1 (1.9)	0.027
Vertigo, n (%)	58 (17.0)	53 (18.4)	5 (9.3)	0.10
Sensory abnormality, n (%)	105 (30.7)	99 (34.4)	6 (11.1)	<0.001
Aphasia, n (%)	54 (15.8)	41 (14.2)	13 (24.1)	0.069
Dysarthria, n (%)	216 (63.2)	183 (63.5)	33 (61.1)	0.73
Dysphagia, n (%)	7 (2.0)	3 (1.0)	4 (7.4)	0.014
Ataxia, n (%)	32 (9.4)	30 (10.4)	2 (3.7)	0.12
**Initial blood pressure**				
SBP (mmHg), median (IQR)	159 (140,180)	161 (140,183)	148 (134,164)	0.007
SBP >140 mmHg, n (%)	249 (72.8)	215 (74.7)	34 (63.0)	0.076
DBP (mmHg), median (IQR)	90 (79,100)	90 (80,101)	90 (78,98)	0.34
eGFR (mL/min), median (IQR)	83 (62,95)	84 (65,96)	74 (50,92)	0.026
**Scoring system**				
Pre-stroke mRS score ≥2, n (%)	31 (9.1)	22 (7.6)	9 (16.7)	0.065
Initial mRS score ≥4, n (%)	261 (76.3)	208 (72.2)	53 (98.1)	<0.001
Initial BI score <40, n (%)	112 (32.7)	74 (25.7)	38 (70.4)	<0.001
Initial GCS score ≤8, n (%)	21 (6.1)	9 (3.1)	12 (22.2)	<0.001
Initial NIHSS score ≥15, n (%)	83 (24.3)	49 (17.0)	34 (63.0)	<0.001
**TOAST classification**				<0.001
Large-artery atherosclerosis, n (%)	104 (30.4)	79 (27.4)	25 (46.3)	
Small-vessel occlusion, n (%)	158 (46.2)	152 (52.8)	6 (11.1)	
Cardioembolism, n (%)	66 (19.3)	44 (15.3)	22 (40.7)	
Other etiology, n (%)	11 (3.2)	10 (3.5)	1 (1.9)	
Undetermined etiology, n (%)	3 (0.9)	3 (1.0)	0 (0.0)	
**Stroke treatment**				
Intravenous thrombolysis (rt-PA), n (%)	47 (13.7)	36 (12.5)	11 (20.4)	0.12
Antiplatelets, n (%)	281 (82.2)	249 (86.5)	32 (59.3)	<0.001
Anticoagulants, n (%)	52 (15.2)	35 (12.2)	17 (31.5)	<0.001
Statins, n (%)	336 (98.2)	286 (99.3)	50 (92.6)	0.007
**Device**				
Nasogastric tube, n (%)	82 (24.0)	32 (11.1)	50 (92.6)	<0.001
Failed WST, n (%)	85 (24.9)	34 (11.8)	51 (94.4)	<0.001
Endotracheal intubation with invasive mechanical ventilation, n (%)	41 (12.0)	14 (4.9)	27 (50.0)	<0.001
Duration of endotracheal intubation (days), median (IQR)	n = 41 12 (5,27)	n = 14 14 (6,39)	n = 27 9 (5,24)	0.22
Non-invasive ventilation, n (%)	9 (2.6)	2 (0.7)	7 (13.0)	<0.001
Retained Foley catheter, n (%)	88 (25.8)	44 (15.3)	44 (81.5)	<0.001
Duration of retained Foley catheter (days), median (IQR)	n = 88 6 (3,13)	n = 44 4 (2,10)	n = 44 8 (5,14)	0.005

BMI was calculated as kg/m^2^

*The control group and pneumonia group were compared using the chi-squared test or Fisher’s exact test. BI, Barthel Index; BMI, body mass index; BPH, benign prostatic hyperplasia; COPD, chronic obstructive pulmonary disease; DBP, diastolic blood pressure; eGFR, estimated glomerular filtration rate; GCS, Glasgow Coma Scale; HIV, human immunodeficiency virus; IQR, interquartile range; mRS, modified Rankin scale; n, number; N, number of men; NIHSS, National Institutes of Health Stroke Scale; rt-PA, recombinant tissue plasminogen activator; SBP, systolic blood pressure; SD, standard deviation; TOAST, Trial of Org 10172 in Acute Stroke Treatment; WST, water-swallowing test.

**Table 2:** Potential predictors of stroke-associated pneumonia in patients with acute ischemic stroke in multivariate analysis.

Variable	Adjusted OR (95% CI)	p-value*
**Demographic**		
Age	1.04 (0.998–1.088)	0.059
**Presenting symptoms**		
Sensory abnormality	0.24 (0.06–1.09)	0.061
Aphasia	0.31 (0.11–1.03)	0.052
**Device**		
Failed WST	87.48 (21.00–364.51)	<0.001
Endotracheal intubation with invasive mechanical ventilation	12.38 (2.44–101.35)	0.001
Retained Foley catheter	5.67 (2.03–15.83)	0.001

The multivariate analysis model was adjusted for age, history of alcohol consumption, smoking, chronic kidney disease, coronary artery disease, atrial fibrillation, weakness, visual disturbance, vertigo, sensory abnormality, aphasia, dysphagia, ataxia, alteration of consciousness, systolic blood pressure >140 mmHg, eGFR, pre-stroke mRS score ≥2, initial mRS score ≥4, initial BI score <40, initial GCS score ≤8, initial NIHSS score ≥15, intravenous thrombolysis (rt-PA), antiplatelets, statins, a failed WST, endotracheal intubation with invasive mechanical ventilation, non-invasive ventilation, and a retained Foley catheter. TOAST classification and patients in whom anticoagulants were used or a nasogastric tube was placed were excluded because of multicollinearity via generalized variance inflation factor criteria >5. The adjusted OR was calculated by logistic regression with a backward method (Wald probability for removal 0.1)

*Control group vs pneumonia group. BI, Barthel index; CI, confidence interval; eGFR, estimated glomerular filtration rate; GCS, Glasgow Coma Scale; mRS, modified Rankin scale; NIHSS, National Institutes of Health Stroke Scale; OR, odds ratio; TOAST, Trial of Org 10172 in Acute Stroke Treatment; WST, water-swallowing test.

### Post-stroke complications, length of stay, discharge status, and 3-month outcomes

The incidence of the following stroke complications was found to be significantly higher during hospitalization in the pneumonia group than in the control group: urinary tract infection, respiratory failure, sepsis, brain edema, asymptomatic hemorrhagic transformation, gastrointestinal bleeding, congestive heart failure, atrial fibrillation with rapid ventricular response, acute kidney injury (AKI), and hyponatremia (**Table 3**). The median hospital stay duration was significantly longer in the pneumonia group than in the control group (10 days vs 3 days; p<0.001). There was a statistically significant between-group difference in discharge status, in that the proportion of patients who died while in the hospital was higher in the pneumonia group than in the control group (20% vs. 3%; p<0.001). Compared with the control group, a significantly higher proportion of patients in the pneumonia group had a 3-month mRS score ≥4 (unable to walk without assistance, bedridden or deceased, 74% vs. 25%, p<0.001) and a 3-month BI score <40 (completely dependent on others; 67% vs. 17%, p<0.001). However, our multivariate analysis did not reveal an independent association between SAP status and a poor 3-month outcome after controlling for premorbid risk factors, age, and stroke severity.

**Table 3:** Post-stroke complications, length of stay, discharge status, and 3-month outcomes.

Variables	Total (n = 342)	Control group (n = 288)	Pneumonia group (n = 54)	p-value*
**Infectious complications**				
Urinary tract infection, n (%)	31 (9)	13 (5)	18 (33)	<0.001
Respiratory failure, n (%)	42 (12)	15 (5)	27 (50)	<0.001
Sepsis, n (%)	64 (19)	17 (6)	47 (87)	<0.001
**Neurological complications**				
Brain edema, n (%)	12 (4)	4 (1)	8 (15)	<0.001
Seizure, n (%)	14 (4)	11 (4)	3 (6)	0.47
Progressive stroke, n (%)	10 (3)	10 (3)	0 (0)	0.37
**Bleeding complications**				
Symptomatic hemorrhagic transformation, n (%)	7 (2)	4 (1)	3 (6)	0.082
Asymptomatic hemorrhagic transformation, n (%)	8 (2)	4 (1)	4 (7)	0.024
Gastrointestinal bleeding, n (%)	11 (3)	4 (1)	7 (13)	<0.001
**Cardiovascular complications**				
Myocardial infarction, n (%)	3 (1)	1 (0)	2 (4)	0.066
Congestive heart failure, n (%)	21 (6)	10 (3)	11 (20)	<0.001
AF with RVR, n (%)	11 (3)	6 (2)	5 (9)	0.018
**Other complications**				
Hypoglycemia, n (%)	5 (1)	4 (1)	1 (2)	0.58
Hyperglycemia, n (%)	3 (1)	2 (1)	1 (2)	0.40
Acute kidney injury, n (%)	21 (6)	6 (2)	15 (28)	<0.001
Hyponatremia, n (%)	9 (3)	4 (1)	5 (9)	0.006
**Length of stay (days), median (IQR)**	3 (2,5)	3 (2,4)	10 (5,16)	<0.001
**Discharge status**				<0.001
Complete recovery, n (%)	10 (3)	10 (3)	0 (0)	
Improvement, n (%)	288 (84)	256 (89)	32 (59)	
No improvement, n (%)	25 (7)	14 (5)	11 (20)	
Death, n (%)	19 (6)	8 (3)	11 (20)	
**3-month stroke outcomes**				
mRS score, median (IQR)	2 (1,4)	2 (1,4)	5 (3,6)	<0.001
mRS score ≥4, n (%)	112 (33)	72 (25)	40 (74)	<0.001
BI score, median (IQR)	95 (35,100)	100 (65,100)	6 (0,55)	<0.001
BI score <40, n (%)	86 (25)	50 (17)	36 (67)	<0.001

*Control group vs pneumonia group; length of stay and treatment outcomes were compared between groups using the Mann–Whitney *U* test and complications using Fisher’s exact test. AF, atrial fibrillation; BI, Barthel index; IQR, interquartile range; mRS, modified Rankin scale; RVR, rapid ventricular response.

### Clinical characteristics of stroke-associated pneumonia

Forty-one (75.9%) of the 54 patients in the pneumonia group had CAP, 13 (24.1%) had HAP, five (9.3%) had VAP, and five (9.3%) had aspiration pneumonia. The median time to develop pneumonia was 1 day (IQR 1, 2). Fever, dyspnea, and cough were the three most common symptoms of pneumonia (**Table 4**). The median number of SIRS criteria met was 3 (IQR 2, 4) and the median CURB-65 score was 2 (IQR 1, 3). Most of the pneumonia lesions observed on radiographs were in the lower lung field (left lower lung, 40.7%; right lower lung, 38.9%). Overall, the most prevalent organism was *Klebsiella pneumoniae*, which was found in 17 of 54 patients (31.5%). When we categorized SAP into CAP and HAP, the most common causative organism in the CAP group was *K*. *pneumoniae*, which was found in 17 of 41 patients (41.5%), whereas carbapenem-resistant *Acinetobacter baumannii* was the most commonly found causative organism in the HAP group at 38.5% (five of 13 patients). **Table 4** also presents the antibiotic susceptibility data, which show that more than 80% of bacterial isolates causing CAP continued to be susceptible to amoxicillin-clavulanic acid, ceftriaxone, and levofloxacin but none of the bacteria causative of HAP were susceptible to meropenem. Antibiotics were prescribed as monotherapy in 77.8% of cases, and the remaining 22.2% of patients with pneumonia received combination treatment. Invasive mechanical ventilation was required in 50.0% of cases and non-invasive ventilation in 13.0%.

**Table 4:** Clinical characteristics and laboratory results of patients with pneumonia.

Clinical variable	Total (n = 54)
** Type of infection**	
• Community-acquired, n (%)	41 (75.9)
• Hospital-acquired, n (%)	13 (24.1)
Ventilator-associated pneumonia, n (%)	5 (9.3)
Aspiration pneumonia, n (%)	5 (9.3)
Time to development of pneumonia after admission (days), median (IQR)	1 (1,2)
**Symptoms and signs of pneumonia**	
• Fever, n (%)	51 (94.4)
• Dyspnea, n (%)	51 (94.4)
• Cough, n (%)	47 (87.0)
• Alteration of consciousness, n (%)	42 (77.8)
• Crackles, n (%)	19 (35.2)
• Pleuritic chest pain, n (%)	2 (3.7)
SIRS criteria met, median (IQR)	3 (2,4)
CURB-65 score, median (IQR)	2 (1,3)
**Chest radiographs**	
• Left lower lung infiltration, n (%)	22 (40.7)
• Right lower lung infiltration, n (%)	21 (38.9)
• Bilateral perihilar infiltration, n (%)	5 (9.3)
**Pathogens causative of community-acquired pneumonia (n = 41)**	
• *Klebsiella pneumoniae*, n (%)	17 (41.5)
• Methicillin-susceptible *Staphylococcus aureus*, n (%)	4 (9.8)
• *Haemophilus influenzae*, n (%)	3 (7.3)
• *Moraxella catarrhalis*, n (%)	1 (2.4)
• Normal flora, n (%)	9 (22.0)
**Pathogens causative of hospital-acquired pneumonia (n = 13)**	
• Carbapenem-resistant *Acinetobacter baumannii*, n (%)	5 (38.5)
• Carbapenem-resistant *Klebsiella pneumoniae*, n (%)	2 (15.4)
• Methicillin-resistant *Staphylococcus aureus*, n (%)	2 (15.4)
• *Stenotrophomonas maltophilia*, n (%)	1 (7.7)
• Normal flora, n (%)	3 (23.1)
**Antibiotic susceptibility of community-acquired pneumonia**	
• Amoxicillin-clavulanic acid, n/N (%)	22/23 (95.7)
• Ceftriaxone, n/N (%)	22/24 (91.7)
• Levofloxacin, n/N (%)	22/26 (84.6)
• Piperacillin-tazobactam, n/N (%)	21/21 (100.0)
**Antibiotic susceptibility of hospital-acquired pneumonia**	
• Amoxicillin-clavulanic acid, n/N (%)	0/7 (0.0)
• Ceftriaxone, n/N (%)	0/7 (0.0)
• Ceftazidime, n/N (%)	0/7 (0.0)
• Gentamicin, n/N (%)	2/7 (28.6)
• Amikacin, n/N (%)	7/7 (100)
• Levofloxacin, n/N (%)	2/7 (28.6)
• Piperacillin-tazobactam, n/N (%)	0/7 (0.0)
• Meropenem, n/N (%)	0/7 (0.0)
• Colistin, n/N (%)	5/5 (100.0)
**Treatment (n = 54)**	
Monotherapy, n (%)	42 (77.8)
• Piperacillin-tazobactam, n (%)	15 (27.8)
• Ceftriaxone, n (%)	15 (27.8)
• Ceftazidime, n (%)	7 (13.0)
• Meropenem, n (%)	2 (3.7)
Combination therapy, n (%)	12 (22.2)
**Respiratory support**	
• Invasive mechanical ventilation, n (%)	27 (50.0)
• Non-invasive ventilation, n (%)	7 (13.0)

CURB-65 is an acronym for the following risk factors: confusion, uremia, elevated respiratory rate, low blood pressure, and age 65 years or older. IQR, interquartile range; n, number; N, number of patients who underwent an antimicrobial susceptibility test; SIRS, systemic inflammatory response syndrome.

### Predictors of a poor 3-month outcome in patients with AIS

The poor outcome group (mRS score of ≥4) included 112 (33%) of the 342 AIS patients at 3 months, and the non-poor outcome group included the remaining 230 patients. **Table 5** shows the univariate associations between factors influencing poor outcomes, and **Table 6** shows the results of the multivariate logistic regression analysis. Having hypertension as a comorbidity (aOR 2.87, 95% CI 1.18–6.98, p = 0.020), a pre-stroke mRS score ≥2 (aOR 4.53, 95% CI 1.50–12.72, p = 0.007), an initial BI score <40 (aOR 3.35, 95% CI 1.57–7.16, p = 0.002), a failed WST (aOR 5.04, 95% CI 2.00–12.74, p = 0.001), and brain edema (aOR 20.67, 95% CI 2.10–203.26, p = 0.009) were independent predictors of a poor outcome after 3 months of AIS.

**Table 5:** Factors identified to be potentially associated with a poor 3-month outcome in patients with acute ischemic stroke via univariate analysis.

	All (n = 342)	Non-poor outcome mRS <4 (n = 230)	Poor outcome mRS ≥4 (n = 112)	p-value*
SAP, n (%)	54 (16)	14 (6)	40 (36)	<0.001
Sex				0.37
Male, n (%)	207 (61)	143 (62)	64 (57)	
Female, n (%)	135 (39)	87 (38)	48 (43)	
Age (years), mean ± SD	65±15	61±14	75±13	<0.001
Age ≥70 years, n (%)	141 (41)	64 (28)	77 (69)	<0.001
BMI, mean ± SD	24±5	24±5	23±5	0.018
Obesity (BMI ≥25), n (%)	122 (36)	87 (38)	35 (31)	0.23
**Lifestyle habits**				
Alcohol consumption, n (%)	87 (25)	67 (29)	20 (18)	0.025
Smoking, n (%)	140 (41)	106 (46)	34 (30)	0.005
**Comorbidities**				
Hypertension, n (%)	246 (72)	154 (67)	92 (82)	0.003
Diabetes mellitus, n (%)	101 (30)	71 (31)	30 (27)	0.44
Chronic kidney disease, n (%)	79 (23)	31 (13)	48 (43)	<0.001
Dyslipidemia, n (%)	185 (54)	120 (52)	65 (58)	0.31
Coronary artery disease, n (%)	42 (12)	23 (10)	19 (17)	0.066
Atrial fibrillation, n (%)	62 (18)	26 (11)	36 (32)	<0.001
Cerebrovascular disease, n (%)	52 (15)	30 (13)	22 (20)	0.11
BPH (men only), N (%)	9 (3)	4 (2)	5 (4)	0.16
COPD, n (%)	7 (2)	3 (1)	4 (4)	0.22
**Presenting symptoms**				
Alteration of consciousness, n (%)	59 (17)	17 (7)	42 (38)	<0.001
Headache, n (%)	25 (7)	19 (8)	6 (5)	0.33
Weakness, n (%)	303 (89)	196 (85)	107 (96)	0.005
Facial palsy, n (%)	91 (27)	61 (27)	30 (27)	0.96
Visual disturbance, n (%)	35 (10)	29 (13)	6 (5)	0.038
Vertigo, n (%)	58 (17)	47 (20)	11 (10)	0.014
Sensory abnormality, n (%)	105 (31)	83 (36)	22 (20)	0.002
Aphasia, n (%)	54 (16)	27 (12)	27 (24)	0.003
Dysarthria, n (%)	216 (63)	140 (61)	76 (68)	0.21
Dysphagia, n (%)	7 (2)	1 (0)	6 (5)	0.006
Ataxia, n (%)	32 (9)	27 (12)	5 (4)	0.030
**Initial blood pressure**				
SBP (mmHg), median (IQR)	159 (140,180)	160 (140,180)	157 (140,182)	0.80
SBP >140 mmHg, n (%)	249 (73)	167 (73)	82 (73)	0.91
DBP (mmHg), median (IQR)	90 (79,100)	90 (80,100)	90 (78,100)	0.90
eGFR (mL/min), median (IQR)	83 (62,95)	87 (72,100)	67 (43,86)	<0.001
**Scoring system**				
Pre-stroke mRS score ≥2, n (%)	31 (9)	8 (3)	23 (21)	<0.001
Initial mRS score ≥4, n (%)	261 (76)	153 (67)	108 (96)	<0.001
Initial BI score <40, n (%)	112 (33)	37 (16)	75 (67)	<0.001
Initial GCS score ≤8, n (%)	21 (6)	4 (2)	17 (15)	<0.001
Initial NIHSS score ≥15, n (%)	83 (24)	24 (10)	59 (53)	<0.001
**TOAST classification**				<0.001
Large-artery atherosclerosis, n (%)	104 (30)	55 (24)	49 (44)	
Small-vessel occlusion, n (%)	158 (46)	132 (57)	26 (23)	
Cardioembolism, n (%)	66 (19)	30 (13)	36 (32)	
Other etiology, n (%)	11 (3)	10 (4)	1 (1)	
Undetermined etiology, n (%)	3 (1)	3 (1)	0 (0)	
**Stroke treatment**				
Intravenous thrombolysis (rt-PA), n (%)	47 (14)	30 (13)	17 (15)	0.59
Antiplatelets, n (%)	281 (82)	199 (87)	82 (73)	0.003
Anticoagulants, n (%)	52 (15)	26 (11)	26 (23)	0.004
Statins, n (%)	336 (98)	226 (98)	110 (98)	1.00
**Device**				
Nasogastric tube placement, n (%)	82 (24)	19 (8)	63 (56)	<0.001
Failed WST, n (%)	85 (25)	22 (10)	63 (56)	<0.001
Endotracheal intubation with invasive mechanical ventilation, n (%)	41 (12)	12 (5)	29 (26)	<0.001
Endotracheal intubation period (days), median (IQR)	12 (5,27)	8 (3,11)	12 (6,32)	0.077
Non-invasive ventilation, n (%)	9 (3)	0 (0)	9 (8)	<0.001
Retained Foley catheter, n (%)	88 (26)	32 (14)	56 (50)	<0.001
Retained Foley catheter interval (days), median (IQR)	6 (3,13)	3 (2,7)	7 (5,16)	<0.001
**Post-stroke infection**				
Urinary tract infection, n (%)	31 (9)	7 (3)	24 (21)	<0.001
Respiratory failure, n (%)	42 (12)	12 (5)	30 (27)	<0.001
Sepsis, n (%)	64 (19)	16 (7)	48 (43)	<0.001
**Post-stroke neurological complications**				
Brain edema, n (%)	12 (4)	2 (1)	10 (9)	<0.001
Seizure, n (%)	14 (4)	3 (1)	11 (10)	<0.001
**Post-stroke bleeding complications**				
Symptomatic hemorrhagic transformation, n (%)	7 (2)	0 (0)	7 (6)	<0.001
Asymptomatic hemorrhagic transformation, n (%)	8 (2)	4 (2)	4 (4)	0.45
Gastrointestinal bleeding, n (%)	11 (3)	2 (1)	9 (8)	<0.001
**Post-stroke cardiovascular complications**				
Myocardial infarction, n (%)	3 (1)	0 (0)	3 (3)	0.034
Congestive heart failure, n (%)	1 (0)	0 (0)	1 (1)	0.33
AF with RVR, n (%)	11 (3)	3 (1)	8 (7)	0.007
**Other complications**				
Hypoglycemia, n (%)	5 (1)	3 (1)	2 (2)	0.66
Hyperglycemia, n (%)	3 (1)	2 (1)	1 (1)	1.00
Acute kidney injury, n (%)	21 (6)	2 (1)	19 (17)	<0.001

*Non-poor outcome group vs poor outcome group with comparisons made using the chi-squared test or Fisher’s exact test. BMI was calculated as kg/m^2^. AF, atrial fibrillation; BI, Barthel Index; BMI, body mass index; BPH, benign prostatic hyperplasia; COPD, chronic obstructive pulmonary disease; DBP, diastolic blood pressure; eGFR, estimated glomerular filtration rate; GCS, Glasgow Coma Scale; mRS, modified Rankin scale; n, number; N, number of male patients; NIHSS, National Institutes of Health Stroke Scale; rt-PA, recombinant tissue plasminogen activator; RVR, rapid ventricular response; SAP, stroke-associated pneumonia; SBP, systolic blood pressure; SD, standard deviation; WST, water-swallowing test.

**Table 6:** Risk factors for a poor outcome after 3 months in patients with acute ischemic stroke identified via multivariate logistic regression analysis.

Variables	Adjusted OR (95%CI)	p-value*
**Comorbidities**		
Hypertension	2.87 (1.18–6.98)	0.020
**Presenting symptoms**		
Alteration of consciousness	2.45 (0.91–6.57)	0.075
**Scoring system**		
Pre-stroke mRS score ≥2	4.36 (1.50–12.72)	0.007
Initial mRS score ≥4	2.96 (0.92–9.53)	0.068
Initial BI score <40	3.35 (1.57–7.16)	0.002
**Device**		
Failed WST	5.04 (2.00–12.74)	0.001
**Post-stroke neurological complications**		
Brain edema	20.67 (2.10–203.26)	0.009
Seizure	6.32 (0.90–44.50)	0.064

*Non-poor outcome group vs poor outcome group. The multivariate analysis model was adjusted for SAP, age, BMI, smoking, hypertension, chronic kidney disease, coronary artery disease, AF, atrial fibrillation, cerebrovascular disease, alteration of consciousness, weakness, visual disturbance, vertigo, sensory abnormality, aphasia, dysphagia, ataxia, eGFR, a pre-stroke mRS score ≥2, an initial mRS score ≥4, an initial BI score <40, an initial GCS score ≤8, an initial NIHSS score ≥15, antiplatelets, a failed WST, endotracheal intubation with invasive mechanical ventilation, non-invasive ventilation, a retained Foley catheter, urinary tract infection, respiratory failure, sepsis, brain edema, seizure, symptomatic hemorrhagic transformation, gastrointestinal bleeding, myocardial infarction, acute kidney injury, hyponatremia, and length of stay. The multivariate analysis model included all factors with a p-value <0.2 in the univariable analysis. TOAST classification and patients in whom anticoagulants were used or a nasogastric tube was placed were excluded because of multicollinearity via generalized variance inflation factor criteria >5. The adjusted OR was calculated by logistic regression with a backward method (Wald probability for removal 0.1). BI, Barthel index; CI, confidence interval; eGFR, estimated glomerular filtration rate; GCS, Glasgow Coma Scale; mRS, modified Rankin scale; NIHSS, National Institutes of Health Stroke Scale; OR, odds ratio; SAP, stroke-associated pneumonia; TOAST, Trial of Org 10172 in Acute Stroke Treatment; WST, water-swallowing test.

## Discussion

Pneumonia is a common stroke complication with a reported frequency of 10%–20% [2–4]. Our finding of a 15.8% incidence of pneumonia in acute stroke patients is within the previously reported range. It is clear that alcohol consumption [43–47] and smoking [48–51] are both risk factors for developing pneumonia. However, according to our univariate analysis, the proportions of alcohol consumption (13.0% vs. 27.8%, p = 0.022) and smoking (27.8% vs. 43.4%, p = 0.032) were lower in the pneumonia group than in the control group. The likelihood of drinking alcohol declined as adults matured into their 70s and 80s. Between 10% and 50% of individuals aged 60 years or older are estimated to consume alcohol, a range that decreases to 20% to 25% in those 75 years or older [52]. Likewise, smoking prevalence is lower among older adults (≥65 years of age; 8.3%) than among younger adults (≤64 years; 22.2%) [53]. According to our univariate data, the mean age of controls was 64 years and that of the pneumonia group was 73 years (p <0.001). The lower percentage of alcohol consumption and smoking in the pneumonia group may be due to the older age of the patients.

Our multivariate analysis revealed that a failed WST, a widely used dysphagia screening tool, was associated with pneumonia (aOR 87.48, 95% CI 21.00–364.51), which is consistent with the data of Sellars et al. (aOR 20.1, 95% CI 10.6–37.8) [4] and Liang et al. (aOR 1.46, 95% CI 1.30–1.65) [54]. Furthermore, a study by Yeh et al. in Taiwan found that screening for dysphagia was associated with a decreased risk of post-stroke pneumonia in all stroke patients (OR 0.42; 95% CI 0.18–1.00, p = 0.05) [55] and concluded that screening for dysphagia could help to prevent pneumonia, emphasizing the importance of swallowing tests in patients with acute stroke.

Tracheal intubation thwarts the cough reflex, compromises mucociliary clearance, injures the tracheal epithelial surface, provides a direct conduit for the rapid transport of bacteria from the upper to the lower respiratory tract, and allows the formation of biofilm on the surface of the endotracheal tube, resulting in an increased risk of pneumonia [56]. Due to the limitation of temporal relations in our study, the relationship between pneumonia and intubation with mechanical ventilation was bi-directional. VAP developed after intubation in 9.3% of all pneumonia patients. The remaining patients may have developed pneumonia before respiratory failure and intubation. A prospective observational study by Hilker et al. found that endotracheal intubation with invasive mechanical ventilation was associated with a significantly increased relative risk of SAP of 7.3 (p<0.001, chi-squared test) [3]. Moreover, a multifactorial analysis in a retrospective study by Sui et al. found that patients who underwent tracheal intubation were 2.980 times more likely to develop SAP than those who did not [9]. Our multivariate analysis showed that endotracheal intubation with invasive mechanical ventilation (aOR 12.38, 95% CI 2.44–101.35) was one of the factors associated with SAP, which is consistent with the findings of the previous studies.

A retained urinary catheter is a well-established risk factor for urinary tract infection [57–59]; it is also a risk factor for post-stroke urinary tract infection in acute stroke patients [60]. Data on the association between Foley catheterization and SAP are limited. One study from the Austrian Stroke Unit Registry in 2016 found that urinary catheterization in hospitalized patients with acute stroke was associated with SAP (aOR 2.42, 95% CI 2.15–2.72) [61], which is consistent with our data (aOR 5.67, 95% CI 2.03–15.83). However, a limitation of our study is that we could not document the temporal relationship between this procedure and the onset of pneumonia. The association between pneumonia and Foley catheter placement could reflect the fact that patients with pneumonia may require urinary catheterization for intensive urine output monitoring, and prolonged bed rest may induce urinary retention [62]. On the other hand, stroke patients with extensive neurological damage also tend to be on bed rest more frequently, which can increase the risk of pneumonia due to the inability to ambulate [63], more frequent catheterization, and prolonged catheterization, which can introduce occult urinary tract infections that may be disseminated into the lung [64]. It is interesting to note that *Klebsiella pneumoniae*, the most common pathogen in our study, was also the second most common in post-stroke urinary tract infection patients [65,66].

In our study, several types of post-stroke complications, including sepsis, urinary tract infection, brain edema, bleeding, cardiovascular events, AKI, and hyponatremia, were more common in the pneumonia group than in the control group (**Table 3**), which is in line with previous reports. Xu et al. detected a higher proportion of sepsis in patients with SAP than in those without SAP (48.4% vs. 17.7%; p<0.001) [67], while Matz et al. found a higher proportion of neurological, cardiac, and other infective complications in patients with SAP than in those without SAP [61]. In terms of outcomes, post-stroke pneumonia has been associated with a significantly longer hospital stay [1,67–75], a higher in-hospital mortality rate [69,76–79], a higher 3-month mortality rate [80], a worse 3-month mRS score [80–83], a worse 14-month mRS score (3.5±1.7 in a SAP group vs. 2.2 in a non-SAP group) [3], and a worse 14-month BI score (50.5±42.4 in a SAP group vs. 81.5±27.8 in a non-SAP group) [3]. These findings agree with our data (**Table 3**) showing that the pneumonia group had a longer hospital stay, poorer discharge status, and worse 3-month stroke outcomes (i.e., worse mRS and BI scores).

Pneumonia is the most common cause of fever within the first 48 hours after an acute stroke [1,7,81,83–85], and pneumonia that occurs within 48 hours of admission is classified as CAP [86]. We found that CAP was present in 75.9% of patients in our pneumonia group; this figure is higher than the value of 58% reported by Hilker et al. [3]. The remaining 24.1% were cases of HAP (**Table 4**). There are limited data on the incidence of VAP in patients who develop SAP. De Montmollin et al. reported that 35.9% of their patients with AIS who required invasive ventilation at admission and had pneumonia during hospitalization had VAP [68]. Furthermore, in the studies reported by Xu et al. [67] and Kasuya et al. [87], 31.9% and 28%, respectively, of cases of pneumonia among patients with acute ischemic or hemorrhagic stroke comprised VAP. In our study, the incidence of VAP among patients with AIS who developed pneumonia was 9.3%, which is lower than the values referenced above. Hemorrhagic stroke is more severe than ischemic stroke [88] and is more likely to disturb the level of consciousness [89]; thus, it may increase the risk of respiratory failure with mechanical ventilation [90]. Moreover, a higher proportion of invasive ventilation is typically associated with an increased risk of VAP [91], which may explain the higher incidence of VAP in the previous studies.

Stroke can impair swallowing function, leading to dysphagia and aspiration [61]. SAP is often caused by aspiration and will usually affect the gravity-dependent portions of the lungs (i.e., the lower lobes) [24,92]. Most of the chest radiographs of our patients who developed pneumonia showed unilateral lower lung involvement (**Table 4**). In a study by Chen et al., the most common presentation was a unilateral lung lesion [93], which is compatible with our results. Of note, aspiration pneumonia, as defined in our study, requires a history of macroaspiration, which accounted for only 9.3% of all cases of SAP.

Acute post-stroke pneumonia often occurs in recently hospitalized patients, and the microbiology does not resemble the CAP commonly caused by *Streptococcus pneumoniae* [94,95]. This etiology of SAP often includes aerobic gram-negative bacteria such as *K*. *pneumoniae*, *Acinetobacter*, *Enterobacter*, *Escherichia coli*, and *Pseudomonas aeruginosa* [92]. In the present study, we found *K*. *pneumoniae* to be the most common pathogen in patients with CAP (41.5%). Studies of SAP by Chen et al., Xu et al., and Guo et al. also found *K*. *pneumoniae* to be the most common pathogenic organism [67,93,96], and this bacterium has also been linked with aspiration pneumonia [11,97–100]. For HAP, the most common causative organisms were carbapenem-resistant *A*. *baumannii* (38.5%) followed by carbapenem-resistant *K*. *pneumoniae* (15.4%), methicillin-resistant *Staphylococcus aureus* (15.4%), and *S*. *maltophilia* (7.7%). These findings are similar to the etiology of HAP and VAP found in other tertiary hospitals in Thailand [101,102].

In our study, we classified antibiotic susceptibility into two categories, namely, CAP and HAP. In CAP, even though *K*. *pneumoniae* was the most prevalent organism, ceftriaxone, levofloxacin, and amoxicillin-clavulanic acid were effective in over 80% of cases (**Table 4**). Therefore, for CAP after a stroke, amoxicillin-clavulanic acid, ceftriaxone, and levofloxacin are still good choices for empirical therapy. In contrast, only 28.6% of the organisms in patients with HAP were susceptible to levofloxacin and gentamicin. Moreover, none of them were susceptible to amoxicillin-clavulanic acid, ceftriaxone, ceftazidime, piperacillin-tazobactam, and meropenem, as shown in **Table 4**. The susceptibility of each organism to meropenem was 87.5%–100% in a retrospective study of HAP among patients with AIS in Pakistan published in 2021 [103]. This difference indicates a trend of meropenem resistance in our region. Thus, combination therapy can be considered for empirical treatment in patients with hospital-onset pneumonia after a stroke.

Several factors have previously been associated with poor outcomes in AIS patients, such as age, stroke severity, dementia, atrial fibrillation, cancer, malnutrition, previous stroke, and heart failure [104–107]. SAP has been found to be associated with higher odds of a long length of stay (OR 1.93 [1.67–2.22]) and a worse functional outcome (OR 7.17 [5.44–9.45]) [108]. SAP also had a high mortality rate [109,110]. In our study, the univariate analysis found SAP to be a significant factor associated with a poor outcome. Unfortunately, it was not an independent risk factor in the multivariate analysis. While this present data does not definitively demonstrate a causative association between SAP and a poor outcome, the various limitations of the study (sample size, retrospective chart review) may have limited the power to detect such an association.

In observational studies, both extremely high and low blood pressure values have been associated with poor outcomes, whether defined by early neurological deterioration, stroke recurrence, death, or late dependency [111–113]. Willmot et al. found that high blood pressure in acute ischemic stroke is associated with subsequent death, death or dependency, and death or deterioration [114]. In our data, having hypertension as a comorbidity was also associated with a poor outcome (aOR 2.87, 95% CI 1.18–6.98, p = 0.020).

According to Golda et al.’s 2020 study, AIS patients with a pre-stroke mRS score ≥2 and mechanical thrombectomy might have an extremely poor prognosis after 3 months [115]. Furthermore, Quinn et al. found that every point increase in the pre-stroke mRS of acute stroke patients is associated with higher mortality at 7 days and 1 year, length of stay, discharge destination, and post-stroke complications of pneumonia and urinary tract infection [116]. Our data found that a pre-stroke mRS score ≥2 was a predictor of poor outcomes (aOR 4.53, 95% CI 1.50–12.72, p = 0.007), which fits with both reports.

We found an initial BI score <40 to be a risk factor for poor outcomes at 3 months (aOR 3.35, 95% CI 1.57–7.16, p = 0.002), which was consistent with data from Saksathien et al. showing that admission BI scores below 50 in AIS patients were correlated with poor 6-month functional outcomes [117]. Moreover, Wade’s follow-up study revealed that the lower the baseline BI score, the higher the death rate among stroke patients 6 months later [118], and Li’s study revealed that BI scoring is a highly valuable scoring system for the mortality risk prediction of patients with acute cerebral infarction [119].

Following the multivariate logistic regression analysis of Smithard’s study in 1996, the presence of an abnormal swallow on bedside assessment after acute stroke remained a significant predictor of mortality (χ2 [1 df] = 6.4, p = 0.01) [120], and multinomial logistic regression in 2007 by the same author showed that residence in a nursing home was more likely to occur in those who failed a WST during the first week of their stroke at 3 months (relative risk ratio [RRR] = 1.73, 95% CI 1.02–2.95), 4 years (RRR 3.35, 95% CI 1.37–8.19), and 5 years (RRR 3.06, 95% CI 1.06–8.83); there was also a significant association with increased mortality at 3 months (RRR 2.03, 95% CI 1.12–3.67) [121]. Congruent with our data, a failed WST (aOR 5.04, 95% CI 2.00–12.74, p = 0.001) was associated with a poor 3-month outcome.

Cerebral edema is a common complication of acute ischemic stroke that leads to poorer functional outcomes and substantially increases the mortality rate [122]. Battey et al. discovered that the presence of swelling independently predicted a worse 3-month outcome in non-lacunar ischemic stroke (mRS ≥3, odds ratio [OR] 4.55, 95% CI 1.21–18.9, p<0.02) [123]. McKeown et al. have shown that a midline shift greater than 3 mm after ischemic stroke can independently predict poor 3-month outcomes (mRS ≥4, OR 4.46, 95% CI 3.56–5.59, p<0.001) [124]. Our study also reiterates that brain edema is a poor prognostic factor (mRS ≥4, aOR 20.67, 95% CI 2.10–203.26, p = 0.009).

This study has several limitations. First, although the total number of patients was not small, the number of patients with pneumonia was relatively small. Second, the study data were collected retrospectively, which means that temporal relationships were difficult to assess. Third, the study was performed on patients from one center, which lacks the scientific rigor or external validity required to support widespread changes and may limit the generalizability of our findings.

## Conclusions

This study confirms that SAP is associated with a failed WST, endotracheal intubation with invasive mechanical ventilation, and placement of a Foley catheter in hospitalized patients with AIS. Patients with SAP had more post-stroke complications, a longer hospital stay, a worse discharge status, and a poor stroke outcome at 3 months. Most of our patients with SAP who developed pneumonia did so within 48 hours of admission and had a unilateral lower lung lesion. The most common causative pathogen in these patients was *K*. *pneumoniae*. In terms of antibiotic susceptibility, amoxicillin-clavulanic acid, ceftriaxone, and levofloxacin can still be recommended as empirical therapy for patients with community-onset SAP. However, combination therapy might be considered for patients with hospital-onset SAP according to the local antibiogram. Finally, having hypertension as a comorbidity, a pre-stroke mRS score ≥2, an initial BI score <40, a failed WST, and brain edema were emphasized as determinants of a poor 3-month prognosis in AIS patients.

## Abbreviations

baumannii: *Acinetobacter baumannii*
ADL: activities of daily living
AIS: Acute Ischemic Stroke
aOR: adjusted Odds Ratio
BI: Barthel Index
BPH: Benign Prostatic Hyperplasia
CI: Confidence Interval
CIOMS: Council for International Organizations of Medical Sciences
DBP: Diastolic Blood Pressure
E. coli: *Escherichia coli*
eGFR: estimated Glomerular Filtration Rate
GCS: Glasgow Coma Scale
ICH-GCP: International Conference on Harmonization-Good Clinical Practice
IQR: interquartile range
K. pneumoniae: *Klebsiella pneumoniae*
ml/min: milliliter per minute
mg/dL: milligram per deciliter
mmHg: millimeters of mercury
mRS: modified Rankin Scale
n: number
NG tube: Nasogastric tube
NIHSS: National Institutes of Health Stroke Scale
qSOFA: quick Sepsis-related Organ Failure Assessment
rt-PA: recombinant tissue Plasminogen Activator
SAP: Stroke-Associated Pneumonia
SBP: Systolic Blood Pressure
SD: Standard Deviation
S. maltophilia: *Stenotrophomonas maltophilia*
SIRS: systemic inflammatory response syndrome
SPSS: Statistical Package for the Social Sciences
TOAST: Trial of ORG 10172 in Acute Stroke Treatment
UTI: Urinary Tract Infection
USA: United States of America
WST: Water-Swallowing Test
